# White Matter Injury Due to Experimental Chronic Cerebral Hypoperfusion Is Associated with C5 Deposition

**DOI:** 10.1371/journal.pone.0084802

**Published:** 2013-12-30

**Authors:** Qinghai Liu, Shuhan He, Leonid Groysman, David Shaked, Jonathan Russin, Steven Cen, William J. Mack

**Affiliations:** 1 Zilkha Neurogenetic Institute, Keck School of Medicine, University of Southern California, Los Angeles, California, United States of America; 2 Department of Neurosurgery, Keck School of Medicine, University of Southern California, Los Angeles, California, United States of America; 3 Department of Preventative Medicine, Keck School of Medicine, University of Southern California, Los Angeles, California, United States of America; University of Leicester, United Kingdom

## Abstract

The C5 complement protein is a potent inflammatory mediator that has been implicated in the pathogenesis of both stroke and neurodegenerative disease. Microvascular failure is proposed as a potential mechanism of injury. Along these lines, this investigation examines the role of C5 in the setting of chronic cerebral hypoperfusion. Following experimental bilateral carotid artery stenosis, C5 protein deposition increases in the corpus callosum over thirty days (p<0.05). The time course is temporally consistent with the appearance of white matter injury. Concurrently, systemic serum C5 levels do not appear to differ between bilateral carotid artery stenosis and sham-operated mice, implicating a local cerebral process. Following bilateral carotid artery stenosis, C5 deficient mice demonstrate decreased white matter ischemia in the corpus callosum when compared to C5 sufficient controls (p<0.05). Further, the C5 deficient mice exhibit fewer reactive astrocytes and microglia (p<0.01). This study reveals that the C5 complement protein may play a critical role in mediating white matter injury through inflammation in the setting of chronic cerebral hypoperfusion.

## Introduction

Cognitive impairment and dementia are disabling conditions that are increasingly common with advancing age. With a rapidly aging population, illnesses such as Alzheimer’s disease (AD) and vascular dementia are significant health-care concerns. Clinical imaging, epidemiology, and pharmacotherapy studies have established a strong association between cortical hypoperfusion and cognitive impairment[1-4]. Experimental and clinical studies have demonstrated that sustained reductions in cerebral blood flow result in white matter ischemic injury[5,6]. Inflammatory upregulation and progressive microvascular failure are critical mediators of acute stroke and cerebral ischemia[7]. In the setting of chronic cerebral hypoperfusion (CCH), sustained inflammation may result in regional perfusion deficits and deficient flow maintenance to critical white matter tracts. 

The complement cascade is a phylogenetically ancient constituent of the innate immune system[8-10]. Its functions are mediated through the sequential activation and proteolytic cleavage of a series of serum proteins. Complement activation occurs through three distinct pathways, all of which converge to activate the fifth (C5) complement component. This activation initiates assembly of the terminal membrane attack complex (C5b-9) (MAC) on target cell surfaces and generation of small C5 cleavage fragments such as C5a (9 kDa) known as anaphylatoxins[11]. The anaphylatoxins affect inflammation through release of cytokines, upregulation of adhesion molecules and increase in vascular permeability[12,13]. A central and critical position in the complement cascade renders C5 (188 kDa) a potent effector of the inflammatory response. The objective of this study is to dissect the role of the C5 complement component in the setting of experimental CCH using an antibody to detect the C5α subunit (125 kDa) of C5 complement[14]. First, the temporal pattern of C5 deposition is examined. Next the effects of C5 modulation on white matter injury and cellular reactivity are assessed.

## Materials and Methods

### Animals and Microcoils

This study was carried out in strict accordance with the recommendations in the University of Southern California Animal Care and Use Committee guidelines. The protocol was approved by the Committee on the Ethics of Animal Experiments of the University of Southern California (IACUC number 11565). All surgery was performed under intraperitoneal ketamine/ xylazine, and all efforts were made to minimize suffering. Wild type C57BL/6J, C5 deficient C57BL/10SnJ 10.D2-Hc^°^ (C5D), and C5 sufficient C57BL/10SnJ 10.D2-Hc^1^ (C5S) mice were purchased from Jackson Laboratories. Studies were performed on male mice between 9-11 weeks old (weighing 24-29g) during the 9am-3pm time period. In an effort to quantify pain, distress, or suffering, we used a 12 point scoring system (0-3 points/category) that included body weight change, physical appearance, unprovoked behavior, and response to external stimuli. Animals were monitored and scores assigned hourly during the perioperative period and daily subsequently until 30 days in the post-operative period. Animals scoring a total of 9 or higher on this scale (out of 12), or 3 in any of the criteria at 24-72h, were considered distressed and euthanized with ketamine/ xylazine. All animals were housed and experimental procedures performed under USC department of Animal Resource guidance. Microcoils (inner diameter: 0.18mm) were purchased from Sawane Spring Co (Sawane, Japan). 

### Bilateral Carotid Artery Stenosis Procedure

Bilateral Carotid Artery Stenosis (BCAS) procedures were performed in accordance with prior publications[15,16]. After a seven day quarantine period, mice were anesthetized with and placed in the prone position. A Laser Doppler Flowmetry (LDF) microtip fiber probe was fixed to the skull at 1 mm posterior and 2mm left of the bregma. The mouse was then rotated to the supine position. Through a midline cervical incision, both common carotid arteries were exposed and a micro-coil (0.18 mm diameter) was applied to each. Rectal temperature was maintained between 36.5C° and 37C°. Sham operated animals underwent the same procedure, except the microcoils were not placed. CBF values were recorded in the supine position just prior to surgery, following application of the first microcoil, and following application of the second microcoil using a Probe 418-1 master probe/ PF 5010 laser Doppler Perfusion Monitoring Unit (Perimed AB, Sweden). Unless otherwise stated, mice were humanely euthanized at the prespecified time points by performing a cardiectomy with saline perfusion while under anesthesia with ketamine (80 mg/kg IP) and xylazine (10 mg/kg IP).

### C5 Deposition and Timeline Studies

C57 Black 6J mice were used for the C5 deposition and timeline studies. Mice underwent either BCAS or sham procedures.

#### Effects of BCAS on C5 Protein Deposition in the Whole Brain and Corpus Callosum

On postoperative day thirty, BCAS (n=6) and sham (n=6) mice were sacrificed. Whole brain samples were harvested for three mice in each cohort. Corpus callosum tissue samples were dissected en bloc for the other three mice in each cohort. Corpus callosum samples were homogenized together [Pierce RIPA buffer (Thermo Scientific, prod #89901) with added protease inhibitor cocktail tablets (Roche, complete mini)] into a single sample for each cohort due to the relatively small quantity of tissue obtained from each animal. Western blot analysis was performed on each sample. Tissue homogenates were separated by SDS-PAGE and transferred to PVDF membranes. Membranes were blocked by 5% non-fat dry milk in TBST (Tris buffered saline, 0.1% Tween 20) for 1 hour, followed by incubation in goat anti-C5α (1:200, Santa Cruz sc-21941) primary antibody overnight at 4°C for detection of the 125 kDa α chain(C5α) of the complement C5 protein (188 kDa). Membranes were then washed with TBST, adding a secondary antibody at room temperature for 1 hour: Donkey anti goat IgG-HRP (1:2000, sc-2020). ECL prime western blotting detection reagent was used for 5 minutes and placed under the UVP biospectrum 600 imaging system. C5α and GAPDH protein levels were measured and relative densities were calculated.

#### C5 Time Course Following BCAS in the Whole Brain

BCAS mice were sacrificed on postoperative day 1 (n=3), 15 (n=3) and 30 (n=3). Whole brain samples were harvested. Western blot analysis was performed according to the above protocol.

#### Serum C5 levels Following BCAS

Serum was obtained by direct cardiac puncture at the time of sacrifice from BCAS (n=9) and sham (n=9) mice used for the above western blot analysis (postoperative day 1, 15 and 30, n=3 in each cohort). ELISA was performed for complement component C5 (ηg C5 protein/ mg total protein) according to the manufacturer’s instructions (Kamiya, WA, USA). 

### C5 Modulation Studies

#### Circle of Willis Anatomy

To date, BCAS studies have only been reported on C57 Black 6J mice. To assess for similarity in configuration of the posterior communicating arteries across the different murine strains utilized in this study, the circle of Willis anatomy of the C5D and C5S mice were examined. No BCAS or sham procedures were performed for this portion of the study. 4 Mice in each of the three groups (C57 black J6, C5D and C5S) were anesthetized using intraperitoneal sodium pentobarbital (50mg/kg). Transcardiac perfusion was performed with 20ml of heparinized saline injection with 50% solution of ink at a pressure of 150mm Hg into the left ventricle until the animal’s tongue, lips and paws turned black. Once the animal was sacrificed, the brain was fixed in 4% PFA from 3 to 4 days and the brain harvested. The circle of Willis vasculature was examined using a Nikon stereomicroscope and photographed. Posterior communicating artery size was visually inspected across the three groups. 

#### Effects of C5 Deficiency in the Setting of BCAS

C5D and C5S mice were used for the C5 modulation experiments. Mice underwent either BCAS or sham procedures and were sacrificed on post-operative day 30. Mice were divided at random into four groups and we aimed for 9-10 mice in each group. We began with n=12 C5D/ BCAS, n=12 C5D/ sham, n=11 C5S/ BCAS, n=9 C5S/ sham and ended with the final group numbers as follows: n=10 C5D/ BCAS, n=10 C5D/ sham, n=9 C5S/ BCAS, n=9 C5S/ sham. On postoperative day 30, the mice were deeply anesthetized with ketamine and xylazine and perfused transcardially with phosphate buffered saline (PH 7.4) to clear the vasculature of blood, then with a fixative containing 4% paraformaldehyde and 0.2% picric acid in 0.1mol/L phosphate buffer (PH7.4). The brains were excised, and stored for an additional 24 hours in paraformaldehyde at 4C°, then in 20% sucrose in 0.1mol/L PBS (PH7.4). The operator was blinded to the strain (C5D/C5S) of the mice and personnel assessing primary outcome were blinded to both the strain (C5D/C5S) and the operative procedure (BCAS/ sham). 

#### Effects of C5 Deficiency in the Setting of BCAS: CBF and Mortality

CBF values are presented as percent change LDF from supine baseline value following placement of the two microcoils. Surgical mortality is defined as death within the first 48 hours after surgery, while postoperative mortality is defined as death between 48 hours of surgery and planned sacrifice (30 days). 

#### Primary Outcome: White Matter Injury in the Medial Corpus Callosum Following BCAS

Harvested brains were embedded in paraffin. A section of the brain located from 1 mm anterior to the bregma to 2 mm posterior to the bregma (adjusted according to mouse atlas) was then sliced into serial 3µm-thick coronal sections. Klüver-barrera (KB) staining was performed on the slice located at the bregma. White matter integrity was evaluated in the medial region of the corpus callosum according to a previously described four point scale at 400x magnification: normal (grade 0), disarrangement of the nerve fibers (grade 1), formation of marked vacuoles (grade 2), and disappearance of myelinated fibers (grade 3)[17]. Number scores were assigned in the left medial corpus callosum and right medial corpus callosum and the two values averaged. Average scores from two independent, blinded observers were calculated for each mouse. The two observers’ scores were then averaged for a final score

#### Immunohistochemical Analysis: Reactive Microglia and Astrocytes, Complement C5 deposition

Harvested brains were embedded in paraffin. A section of the brain located from 1 mm anterior to the bregma to 2 mm posterior to the bregma (adjusted according to mouse atlas) was then sliced into serial 3µm-thick coronal sections. Immunohistochemistry was performed on the slice posterior to the slice used for KB staining according to manufacturer’s instructions. After immunohistochemistry was performed, slides were deparaffined, and then hydrated by a series of different concentration alcohol (from 100% to 70%). Antigen was retrieved by microwave, dipped in 3% H2O2 for 10 min, and then blocked with serum. 

Slides were incubated overnight with a rabbit anti- glial fibrillary acidic protein (GFAP) antibody (diluted 1:10 000; Dako, Denmark), rabbit anti-ionized calcium-binding adapter molecule 1(IBA1) antibody (1:200; Wako, Japan), or rabbit complement component C5α (125kDa) antibody (1:50 Santa Cruz, SC-21941). Subsequently, sections were treated with the appropriate biotinylated secondary antibody Vectastain Elite ABC kit (Vector Laboratories, Burlingame, California, USA) and visualized with diaminobenzidine (DAB). Photos of the immunostained slices were captured by a LAS AF microscope (Leica, Germany). The optical density of DAB signal was analyzed and quantified using NIH Image J software (rsbweb.nih.gov/ij/). The number of positive GFAP and IBA-1 cells were counted in one high powered field in the left medial corpus callosum and right medial corpus callosum and the two values averaged. Likewise, mean density for C5α was measured in one high powered field in the left medial corpus callosum and right medial corpus callosum and the two values averaged. The images were converted to 8 bit and adjusted to threshold to count the positive cells (GFAP/ IBA-1) Protocols followed the NIH Image J user guide.

### Statistical Analysis

SPSS-18 software was used to analyze results. Data were presented as mean±SEM (normally distributed) or median and interquartile range (non-parametric). Two tailed t test and one-way ANOVA followed by Tukey’s test were used to compare continuous variables. Kruskal Wallis test with post hoc pairwise comparisons with Bonferroni adjustment was used to compare ordinal data.

## Results

### Effects of BCAS on C5 Protein Deposition in the Whole Brain and Corpus Callosum

**Figure 1:**
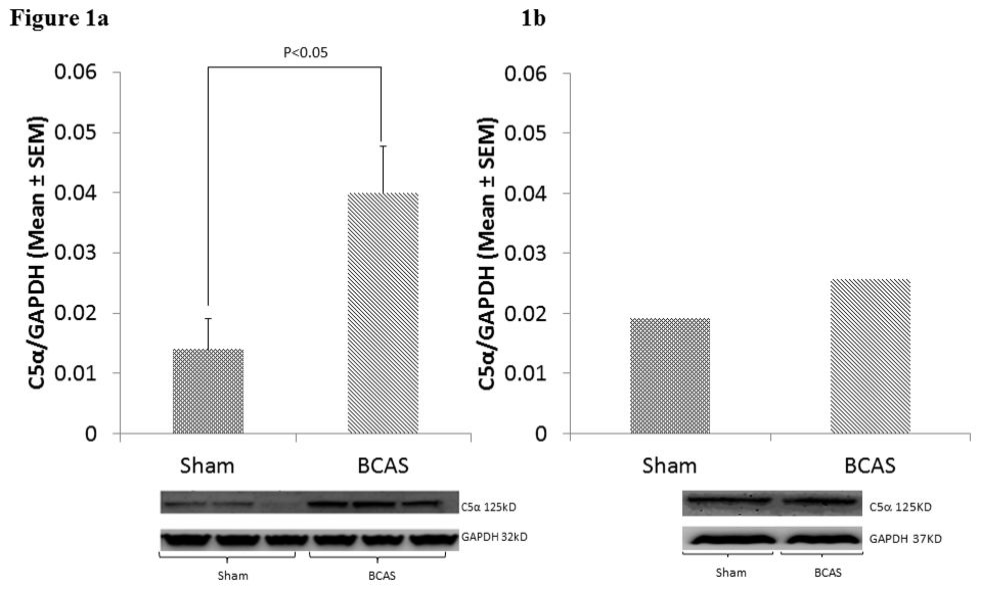
C5 deposition by Western blot Analysis. C5α levels in sham and BCAS mice at thirty days in A) whole brains and B) corpus callosum homogenates. Bottom: Western Blots. Top: Relative C5α densities represented graphically (mean±SEM). Error bars are not presented in Figure 1b, as homogenates of three separate corpus callosum samples are combined into a single sample for each group (due to relatively small amount of tissue obtained from each corpus callosum dissection, n=3 for whole brain, n=3 for corpus callosum homogenates).

Postoperative day 30 western blot analysis of whole brain homogenates demonstrated a C5α/ GAPDH ratio of 0.044 ± 0.02 (n=3) for the BCAS cohort and a ratio of 0.014±0.01 (n=3) for the sham cohort (p<.05). See figure 1a.

Postoperative day 30 western blot analysis of one homogenate from 3 separate BCAS corpus callosum samples demonstrated a C5α/ GAPDH ratio of .0258 while analysis of one homogenate from 3 separate sham corpus callosum samples demonstrated a C5α/ GAPDH ratio of 0.019. See figure 1b. 

CBF values for the total BCAS cohort (mice utilized for analysis of whole brain and corpus callosum, above) demonstrated a CBF change of -26.50 % ± 10.29 (n=6) relative to baseline while sham operated mice exhibited a -5.33% ± 2.14 change (n=6). See Figure S1.

### C5 Time Course Following BCAS in the Whole Brain

**Figure 2:**
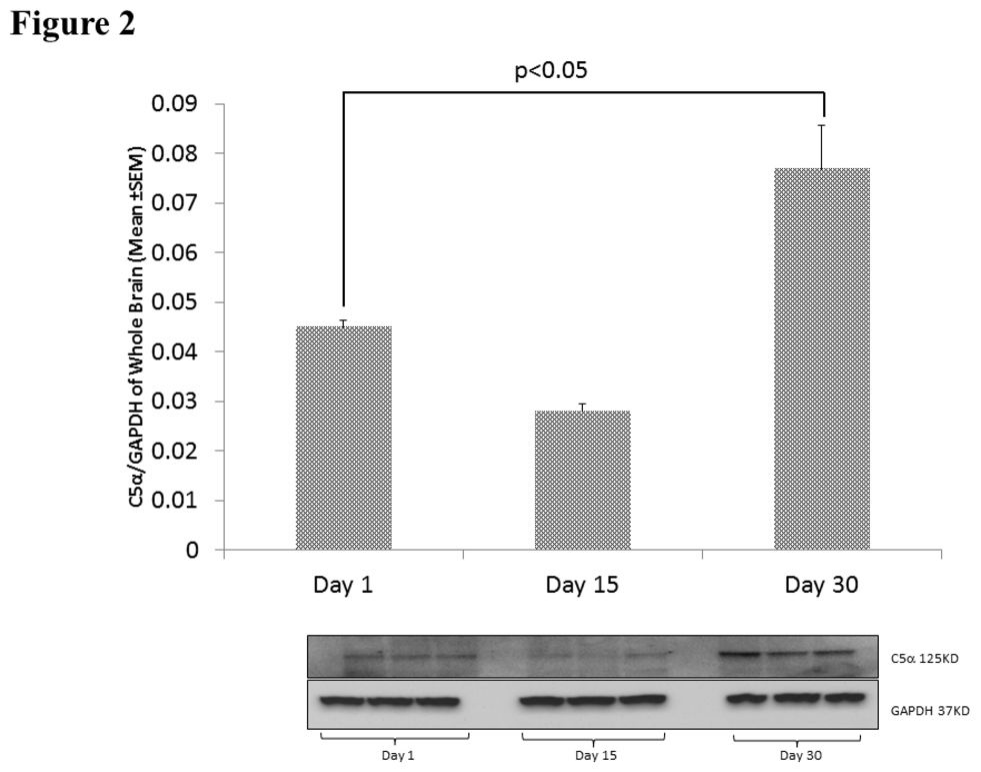
C5 deposition timeline by Western blot Analysis. C5α levels in BCAS mice at day 1 (n=3), 15 (n=3), and 30 (n=3). Bottom: Western Blots. Top: Relative C5α densities represented graphically (mean±SEM).

Western blot analysis yielded mean C5α/ GAPDH ratios of 0.045 ± 0.001 on day 1 (n=3), 0.028±0.001 on day 15 (n=3), and 0.08±0.001 on day 30 (n=3). Day 1 and day 30 values were significantly different (p<0.05). CBF change for the 9 BCAS mice was -38.33% ± 6.37. See figure 2. 

### Serum C5 levels Following BCAS

**Figure 3:**
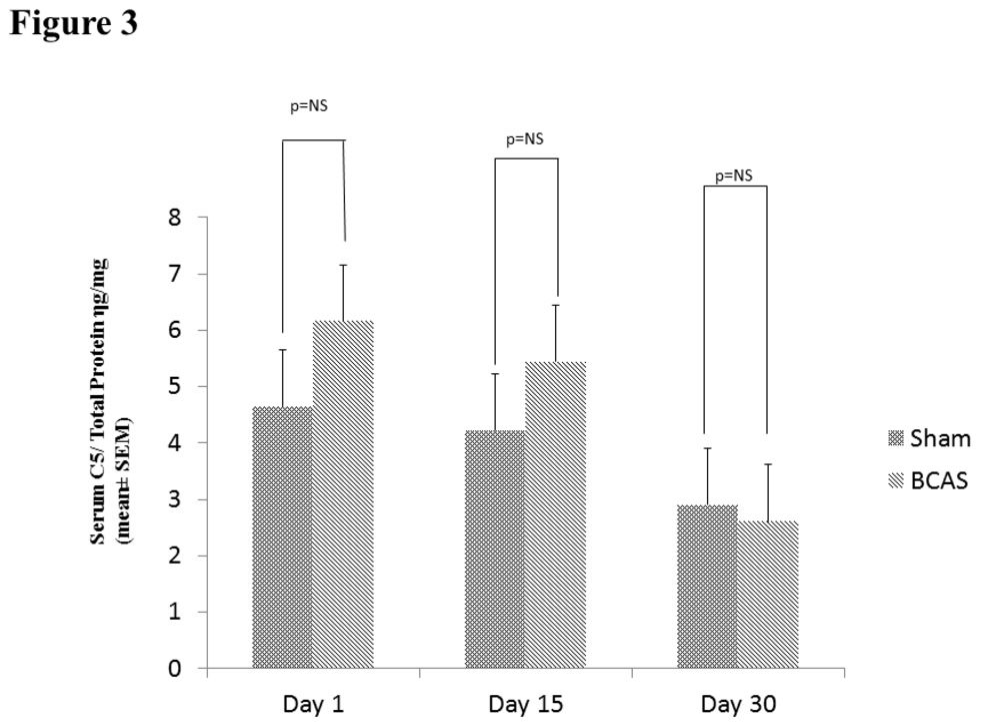
Serum C5 levels. Serum C5 measured by ELISA on day 1 (n=4), 15 (n=3), 30 (n=3) for sham and BCAS mice (mean±SEM).

Serum Elisa C5 values (ηg/mg total protein) were not significantly different between BCAS (day 1: 6.20 ± 2.70, n=4; day 15: 5.40±1.10, n=3; day 30: 2.60±1.10, n=3) and sham operated animals (day 1: 4.70±3.50, n=4; day 15: 4.20±3.80, n=3; day 30: 2.90±01.20, n=3) at any time point. See figure 3.

### Circle of Willis Anatomy

Posterior communicating arteries (PCOM) were present in all mice and the Circle of Willis anatomy did not seem to differ grossly between the C57 black 6J, C5D and C5S strains (n=4 in each group) on visual inspection. See Figure S2.

### Effects of C5 Deficiency in the Setting of BCAS: CBF and Mortality

Mean CBF change in the four cohorts were as follows: C5D sham: -3.71% ±2.49; C5D BCAS: -15.10% ±5.38; C5S sham: -1.00% ±1.00; C5S BCAS -13.00% ±4.29. Surgical mortality in the four cohorts was as follows: C5D sham: 0%; C5D BCAS: 0%; C5S sham 0%; C5S BCAS: 18.2%. One of the animals from the C5D sham group died due to accidental trauma during handling. Post-operative mortality in the four cohorts was as follows: C5D sham: 16.7%; C5D BCAS: 9.1%, C5S sham 0%, C5S BCAS: 0%. See Table S1. Combined surgical and post-operative mortality were previously reported by other groups at 15% for the 0.18mm BCAS group and 0% for the sham operated group[18].

### Primary Outcome: White Matter Injury in the Medial Corpus Callosum Following BCAS

**Figure 4:**
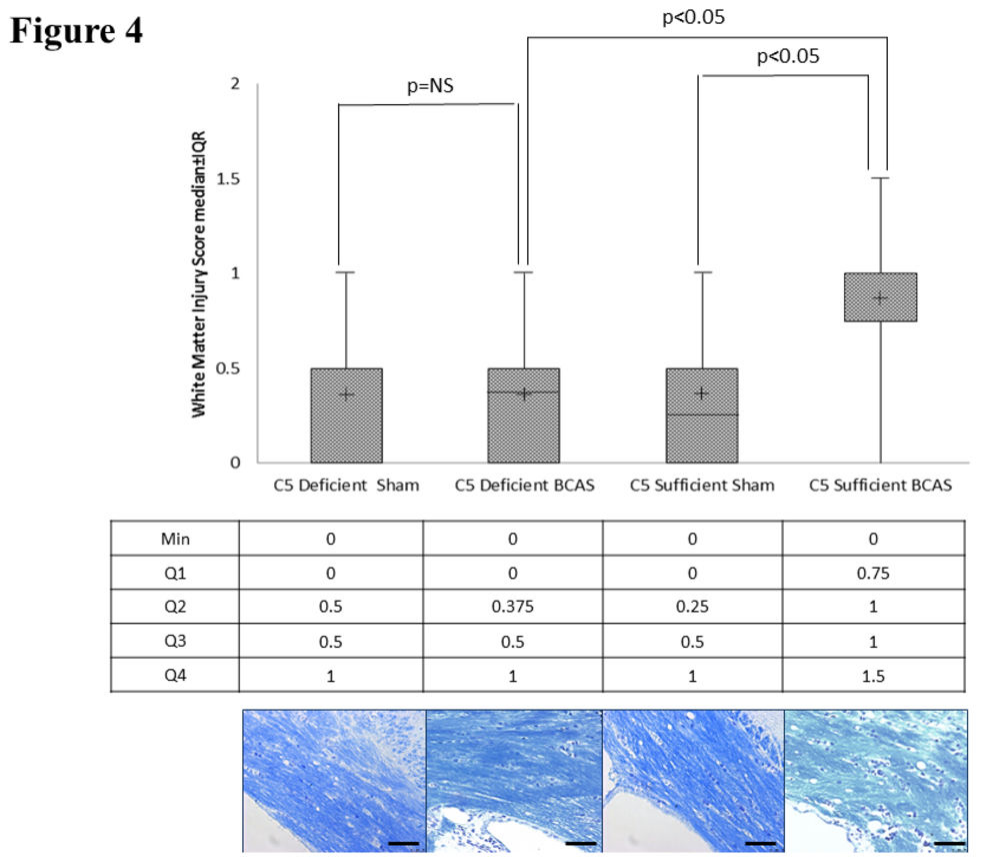
White matter ischemia. Kluver-Barrera staining for white matter ischemic changes in the medial corpus callosum in C5 sufficient and C5 deficient mice subjected to sham and BCAS operations. Above: White matter injury scores. Middle line is the median. The (+) is the mean. The upper and lower lines on the box are the 75% and 25%, respectively. The uppermost and lowermost bars are minimum and maximum, respectively. Values in the text are expressed as median and interquartile range Below: Representative coronal sections of the right corpus callosum with high magnification insert (medial corpus callosum). Bars indicate 50µm. n=10 C5D/ BCAS, n=10 C5D/ sham, n=9 C5S/ BCAS, n=9 C5S/ sham.

The Kruskal Wallis test demonstrated a significant global difference in median white matter injury score among the four groups (C5S BCAS, C5S Sham, C5D BCAS, C5D Sham; p=0.02). The post hoc pairwise comparisons with Bonferroni adjustment showed significant differences in median white matter injury score between C5S BCAS (1.0, IQR:0.75-1.0) and C5S sham mice (0.25, IQR:0-0.5, p<0.05). Further, C5D BCAS mice (0.375, IQR:0-0.5) demonstrated significantly less white matter injury than C5S BCAS mice (1.0, IQR:0.75-1.0, p<0.05). No differences in white matter injury existed between C5D sham (0.5, IQR:0-0.5) and C5D BCAS mice (0.375, IQR:0-0.5, p=ns). See figure 4.

### Immunohistochemical Analysis: Reactive Microglia and Astrocytes, Complement C5 deposition

#### Reactive Microglia: IBA-1

**Figure 5:**
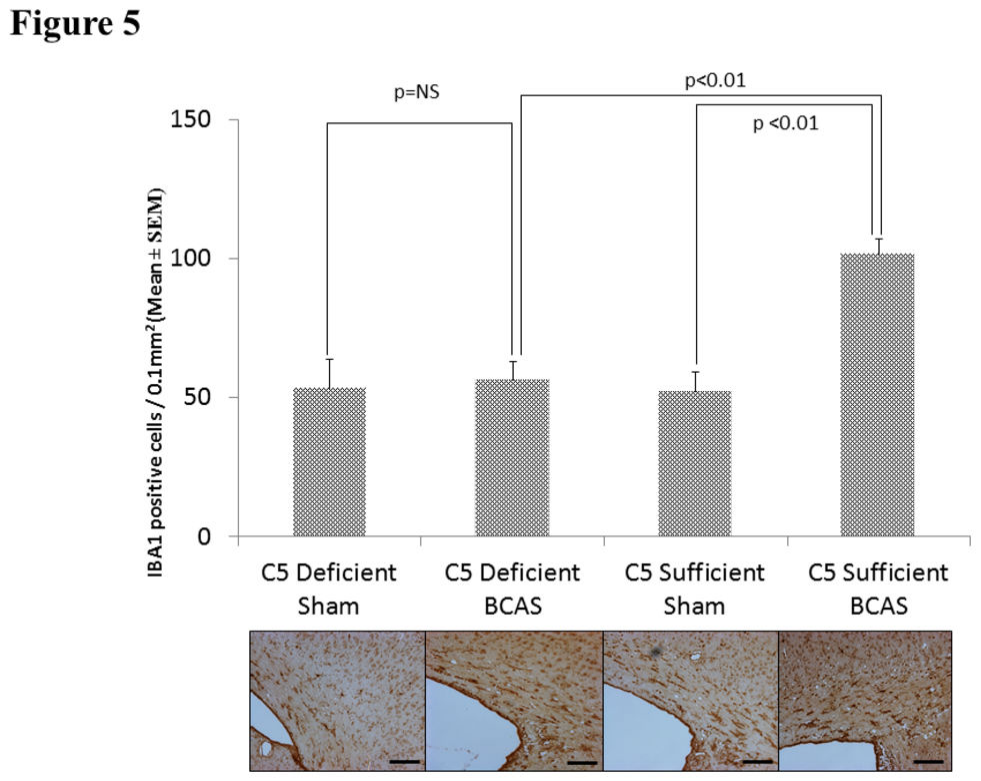
Reactive Microglia: IBA-1 staining for reactive microglia in the medial corpus callosum in C5 sufficient and C5 deficient mice subjected to sham and BCAS operations. Above: IBA-1 positive cell counts in each experimental group. Below: Representative coronal sections of the right corpus callosum with high magnification insert (medial corpus callosum). Bars indicate 50µm. n=10 C5D/ BCAS, n=10 C5D/ sham, n=9 C5S/ BCAS, n=9 C5S/ sham.

There were significant differences in IBA-1 cell count staining between C5S BCAS (101.50 ± 5.71) and C5S sham mice (51.96 ± 7.33, p<0.01) in the corpus callosum. Further, C5D BCAS mice (56.41 ±6.43) demonstrated significantly less IBA-1 staining density than C5S BCAS mice (101.51 ±5.71, p<0.01). No differences in IBA-1 staining density existed between C5D sham (53.31 ±10.51) and C5D BCAS mice (56.41 ± 6.43, p=ns). See Figure 5.

### Reactive Astrocytes: GFAP

**Figure 6:**
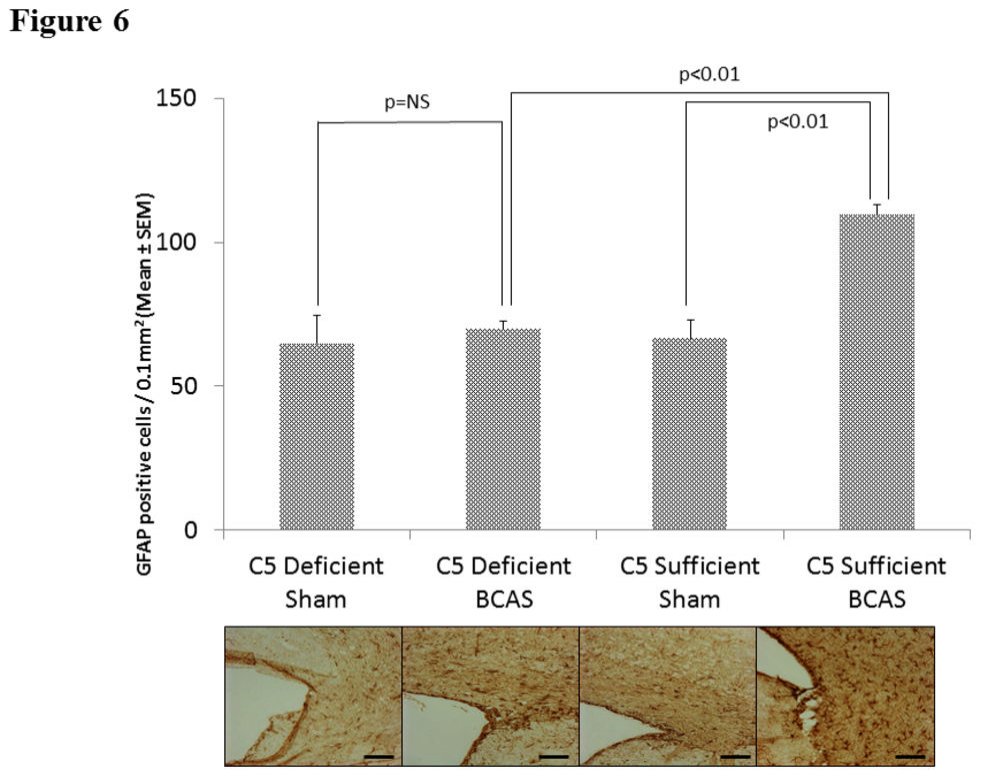
Reactive Astrocytes: GFAP staining for reactive astrocytes in the medial corpus callosum in C5 sufficient and C5 deficient mice subjected to sham and BCAS operations. Above: GFAP positive cell counts in each experimental group. Below: Representative coronal sections of the right corpus callosum with high magnification insert (medial corpus callosum). Bars indicate 50µm. n=10 C5D/ BCAS, n=10 C5D/ sham, n=9 C5S/ BCAS, n=9 C5S/ sham.

There were significant differences in GFAP cell count staining between C5S BCAS (109.33 ± 3.85) and C5S sham mice (66.73 ± 6.41, p<0.01) in the corpus callosum. Further, C5D BCAS mice (69.91 ± 2.58) demonstrated significantly less GFAP staining density than C5S BCAS mice (109.33 ± 3.85, p<0.01). No differences in GFAP staining density existed between C5D sham (64.77 ± 9.69) and C5D BCAS mice (69.91 ± 2.58, p=ns). See Figure 6.

### Complement Deposition: C5α

**Figure 7:**
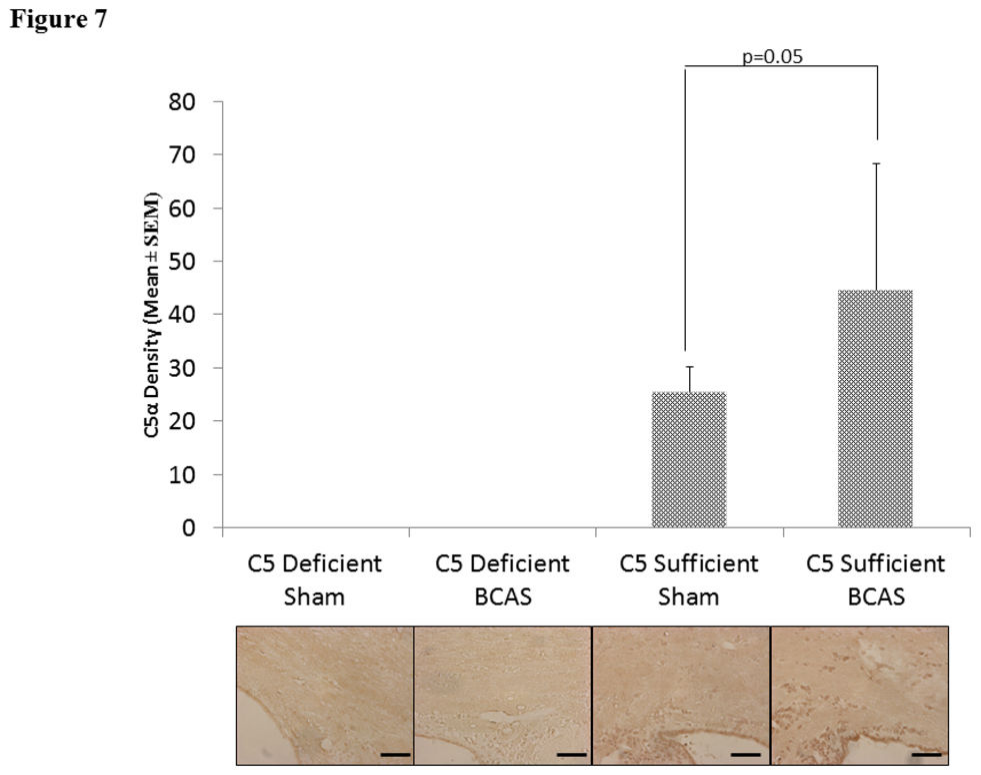
C5 deposition: C5α staining in the medial corpus callosum in C5 sufficient and C5 deficient mice subjected to sham and BCAS operations. Above: C5α density in each experimental group (arbitrary units). Below: Representative coronal sections of the right corpus callosum with high magnification insert (medial corpus callosum). Bars indicate 50µm. n=10 C5D/ BCAS, n=10 C5D/ sham, n=9 C5S/ BCAS, n=9 C5S/ sham.

There were significant differences in C5α density staining between C5S BCAS (44.72 ± 23.60) and C5S sham mice (25.55 ± 4.59, p=0.05) in the corpus callosum. Neither C5D BCAS nor sham mice demonstrated discernible C5α deposition. See Figure 7. There were also significant differences in C5α density staining between C5S BCAS (1099.04 ± 332.60) and C5S sham mice (338.18 ± 76.02, p<0.04 ) in the cerebral cortex.

## Discussion

The pathophysiology of white matter injury following chronic cerebral ischemia is not well understood. Cerebral hypoperfusion involves blood- brain barrier breakdown[19,20] , matrix metalloproteinase activation[21], glial reactivity[17], oligodendroglial apoptosis[22] ,and cytokine/ chemokine upregulation[23]. Evidence suggests that inflammation plays a critical role. The complement cascade is a potent effector of inflammation during physiologic stress. Through the broad pro-inflammatory actions of the anaphylotoxins, complement upregulation is associated with cellular injury in the setting of stroke and neurodegenerative disease[24]. Chronic cerebral hypoperfusion integrates elements of each. 

The temporal course and functional significance of various complement components has been studied extensively in the setting of cerebral ischemia/ reperfusion injury[25]. Specifically, evidence suggests that neurons in the CNS generate C5a following ischemic stress, leading to neuronal apoptosis[26]. Studies have demonstrated ischemic protection in C3 knockout mice [27] and through pharmacologic inhibition of the C3a and C5a receptors[28]. C3a receptor modulation of granulocyte infiltration appears to be reperfusion dependent[29]. Together, these results suggest an anaphylatoxin mediated inflammatory mechanism of complement related injury. The complement cascade also assumes an integral role in the initiation and progression of neurodegenerative disease. Fibrillar Aβ plaques with extensive deposition of multiple complement components are found in brains of AD patients[30-32]. Early complement proteins (C1q, C4, C3, Factor B) have been localized to both amyloid plaques and tangles, while MAC has also been associated with myelin membranes[33]. Prior studies have demonstrated that C5 inhibition results in decreased Aβ plaque load and improved neurological function in the APP mouse model[34]._,_[32] 

The current study examines the function of the C5 protein in white matter ischemic injury following experimental CCH, and is the first to explore the role of the complement system in this disease process. C5 occupies a critical position within the complement cascade, influencing both cellular lysis through generation of the membrane attack complex and inflammatory upregulation via the C5a anaphylotoxin. The data generated from this study advances the hypothesis that the C5 complement protein, through its pro-inflammatory effects promotes microvascular failure in susceptible white matter tracts in the setting of CCH. This microvascular failure may lead to small vessel ischemic injury.

Complement upregulation engenders a pro-inflammatory milieu which initiates microvascular failure and resultant cerebral ischemia[35]. Thrombosis of small blood vessels can result in white matter ischemic change in the same fashion that the no-reflow phenomenon in large vessel stroke promotes recruitment of marginally viable penumbral tissue into the ischemic core through compression of small capillaries[36,37]. In this study, the pattern of C5 upregulation following experimental CCH is temporally concordant with the histopathological damage evidenced at sacrifice on day thirty. These changes are evident on western blot and immunohistochemical analysis. Broad C5 upregulation might suggest a lower susceptibility threshold for white matter, rather than an anatomically specific regional pathology. This is consistent with the characteristic pattern of cerebral hypoperfusion secondary to carotid artery stenosis, which typically causes ischemic changes in watershed vascular territories[38]. Astrocyte and microglial activation within the corpus callosum suggests an inflammatory process. Complement related alterations in the neurovascular/oligovascular units could be the initiating source. Similarity in serum C5 levels between BCAS and sham operated animals at all the time points implicates a process endogenous to the native brain cells, rather than leakage of systemic inflammation through a compromised blood-brain barrier. The findings suggest an association, or a plausible role, for local upregulation of the C5 complement protein. Although the systemic levels of C5 appear to not be significant in sham operated and BCAS mice, the samples sizes were low, so we cannot rule out the possibility that systemic upregulation of C5 could have contributed. However, power analysis for white matter ischemia suggests that 10 animals are enough to demonstrate significant change

To date, the CCH model employed in this investigation has only been validated in C57 black 6J mice. Despite the minimal genetic divergence between murine C57 black 6 and C57 black 10 strains, gross anatomic observations were performed to document the presence of small PCOM arteries (similar to C57 black 6) in the C57 black 10 mice prior to the C5 deficiency experiments. The presence of a large PCOM artery might render the microcoil application incapable of producing changes consistent with CCH secondary to collateral blood flow. Vessel caliber appeared similar between the strains. The C5D BCAS mice showed significantly less injury than the C5S BCAS cohort. Consistent with prior publications, the differences in white matter ischemic change, and number of reactive astrocytes/ microglia were significant between the C5S sham and BCAS (essentially wild-type) cohorts[15,16]. However, The C5D BCAS mice demonstrated no significant white matter ischemic disease or reactivity of astrocytes/ microglia when compared to the C5D sham operated animals. 

An inherent limitation of the study is the use of genetically altered mice. It is possible that as a result of congenital C5 deficiency, these mice could evolve an adaptive process that affects neuroprotection. Assessment of targeted C5 inhibition would help address this question. Also of note, the C5 mutant allele was created by a “TA” 2 base pair deletion on the C5 α complement subunit (125 kDa), resulting in a deficient phenotype, not a genetic knockout[39,40]. Therefore, C5α antibody was utilized to quantify C5 deposition in both the timeline and modulation experiments due to better visualization and binding of the antibody; direct C5a subunit (9 kDa) visualization was not readily available. The absence of C5 staining in the C5D sham and BCAS mice serves a suitable negative control.

Further, this study focuses on the time course of C5 deposition and the histopathology associated with C5 modulation. It will be critical to address the behavioral/ neurocognitive manifestations of these structural changes in future studies. Although C5 occupies a central position in the complement cascade and appears to influence white matter ischemic injury following CCH, the role and contributions of other, upstream complement proteins remains to be determined. 

## Supporting Information

Figure S1 **Representative visual documentation of cerebral hypoxia.** Left: Laser Doppler Flowmetry measurements (arbitrary units) before surgery, after first microcoil, and after second microcoils (separated by vertical lines). Right: Corresponding hypoxyprobe of the coronal section at the bregma.
(TIF)

Figure S2 **Anatomical assessment of Posterior Communicating Arteries.** A) Wild Type C57BL/6J, B) C5 deficient C57BL/10SnJ 10.D2-HC^°^ and C) C5 sufficient C57BL/10SnJ 10.D2-Hc^1^ mice all demonstrate similar size and morphology of posterior communicating arteries anatomy. Arrows point to Posterior Communicating Arteries.
(TIF)

Table S1 **Cerebral blood flow and mortality rates in C5 sufficient and C5 deficient mice after sham and BCAS operations.** Mortality rates are expressed as frequencies and cerebral blood flow values as percentage change from preoperative value (mean ± SEM).
(TIF)
