# Correction: White Matter Injury Due to Experimental Chronic Cerebral Hypoperfusion Is Associated with C5 Deposition

**DOI:** 10.1371/journal.pone.0094516

**Published:** 2014-04-01

**Authors:** The PLOS ONE Staff

Dr. Thomas C. Scotton should be included in the author byline. He should be listed as the sixth author, and is affiliated with Department of Neurosurgery, Keck School of Medicine, University of Southern California, Los Angeles, California, United States.

The contributions of this author are as follows: conceived and designed the experiments, performed the experiments, and analyzed the data.
