# Supplementary figures and images for: White Matter Injury Due to Experimental Chronic Cerebral Hypoperfusion Is Associated with C5 Deposition
Source: PLoS One. 2013 Dec 30;8(12):e84802. doi: 10.1371/journal.pone.0084802 (PMC3875540; doi:10.1371/journal.pone.0084802)

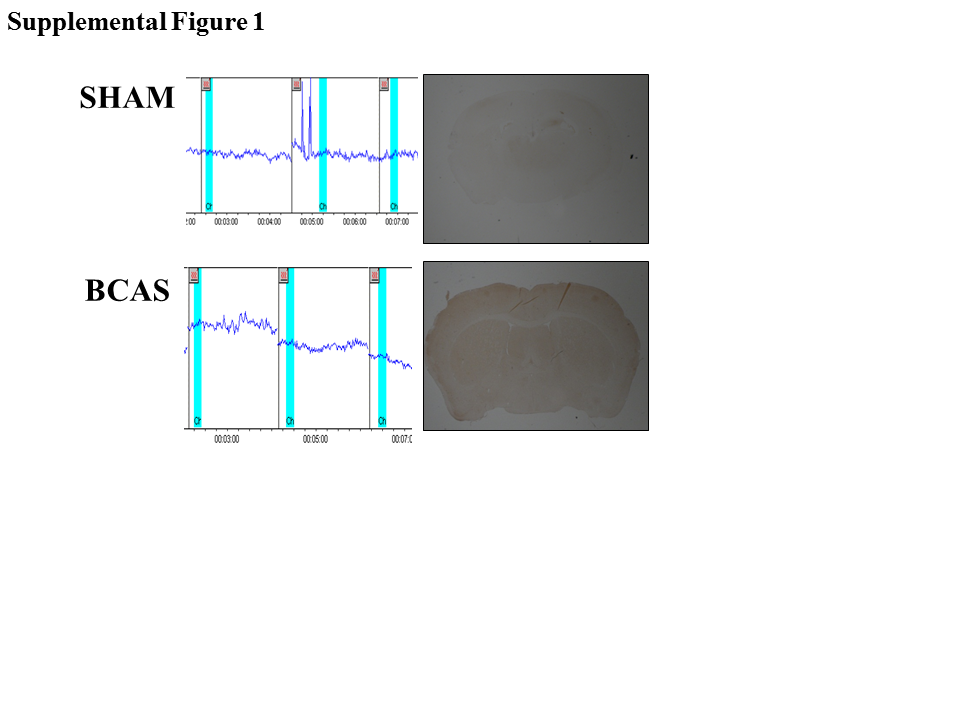

Supplement: Figure S1 — Representative visual documentation of cerebral hypoxia. Left: Laser Doppler Flowmetry measurements (arbitrary units) before surgery, after first microcoil, and after second microcoils (separated by vertical lines). Right: Corresponding hypoxyprobe of the coronal section at the bregma. (TIF) [file pone.0084802.s001.tif]

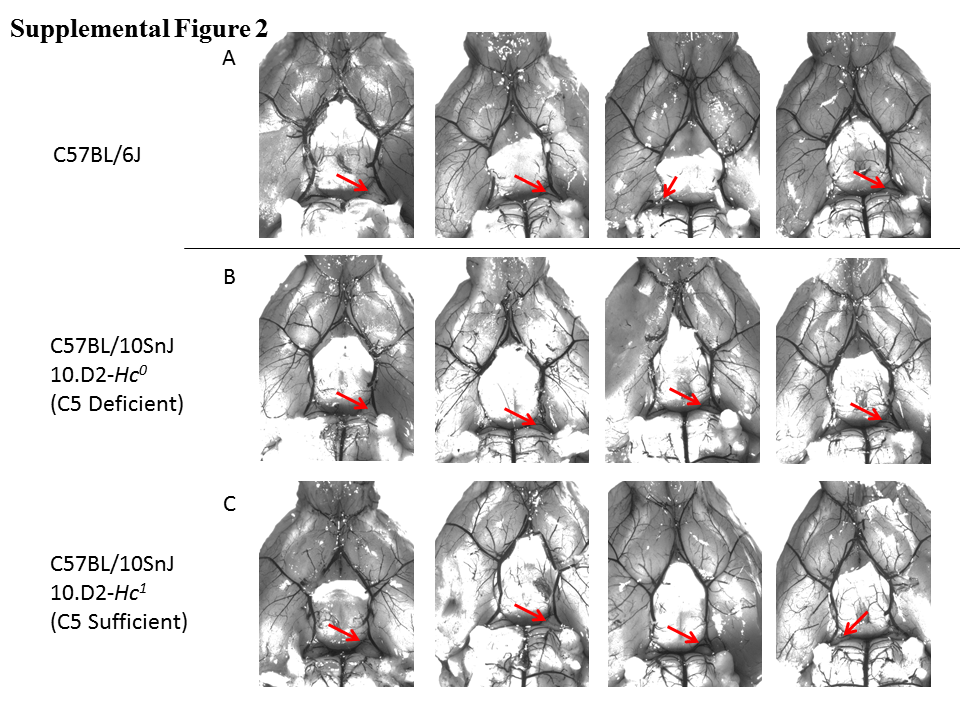

Supplement: Figure S2 — Anatomical assessment of Posterior Communicating Arteries. A) Wild Type C57BL/6J, B) C5 deficient C57BL/10SnJ 10.D2-HC° and C) C5 sufficient C57BL/10SnJ 10.D2-Hc1 mice all demonstrate similar size and morphology of posterior communicating arteries anatomy. Arrows point to Posterior Communicating Arteries. (TIF) [file pone.0084802.s002.tif]
